# IL7R gene expression network associates with human healthy ageing

**DOI:** 10.1186/s12979-015-0048-6

**Published:** 2015-11-11

**Authors:** Willemijn M. Passtoors, Erik B. van den Akker, Joris Deelen, Andrea B. Maier, Ruud van der Breggen, Rick Jansen, Stella Trompet, Diana van Heemst, Evelyna Derhovanessian, Graham Pawelec, Gert-Jan B. van Ommen, P. Eline Slagboom, Marian Beekman

**Affiliations:** Section of Molecular Epidemiology, Leiden University Medical Center, Zone S5-P, P.O. Box 9600, 2300 RC Leiden, The Netherlands; The Delft Bioinformatics Lab, Delft University of Technology, 2600 GA Delft, The Netherlands; Netherlands Consortium for Healthy Ageing, Leiden University Medical Center, P.O. Box 9600, 2300 RC Leiden, The Netherlands; Section of Gerontology and Geriatrics, Department of Internal Medicine, VU University Medical Center, Amsterdam, Netherlands; Department of Psychiatry, VU University Medical Center, Neuroscience Campus Amsterdam, 1081 BT Amsterdam, The Netherlands; Department of Cardiology, Leiden University Medical Center, P.O. Box 9600, 2300 RC Leiden, The Netherlands; Department of Gerontology and Geriatrics, Leiden University Medical Center, P.O. Box 9600, 2300 RC Leiden, The Netherlands; Center for Medical Research, University of Tübingen, 72072 Tübingen, Germany; Center for Human and Clinical Genetics, Leiden University Medical Center, P.O. Box 9600, 2300 RC Leiden, The Netherlands; The Netherlands Center for Medical Systems Biology, Leiden, The Netherlands

**Keywords:** IL7R, Gene expression, Healthy ageing

## Abstract

**Background:**

The level of expression of the interleukin 7 receptor (*IL7R*) gene in blood has recently been found to be associated with familial longevity and healthy ageing. IL7R is crucial for T cell development and important for immune competence. To further investigate the IL7R pathway in ageing, we identified the closest interacting genes to construct an *IL7R* gene network that consisted of *IL7R* and six interacting genes: *IL2RG*, *IL7*, *TSLP*, *CRLF2*, *JAK1* and *JAK3*. This network was explored for association with chronological age, familial longevity and immune-related diseases (type 2 diabetes, chronic obstructive pulmonary disease and rheumatoid arthritis) in 87 nonagenarians, 337 of their middle-aged offspring and 321 middle-aged controls from the Leiden Longevity Study (LLS).

**Results:**

We observed that expression levels within the *IL7R* gene network were significantly different between the nonagenarians and middle-aged controls (*P* = 4.6 × 10^−4^), being driven by significantly lower levels of expression in the elderly of *IL7*, *IL2RG* and *IL7R*. After adjustment for multiple testing and white blood cell composition and in comparison with similarly aged controls, middle-aged offspring of nonagenarian siblings exhibit a lower expression level of *IL7R* only (*P* = 0.006). Higher *IL7R* gene expression in the combined group of middle-aged offspring and controls is associated with a higher prevalence of immune-related disease (*P* = 0.001). On the one hand, our results indicate that lower *IL7R* expression levels, as exhibited by the members of long-lived families that can be considered as ‘healthy agers’, are beneficial in middle age. This is augmented by the observation that higher *IL7R* gene expression associates with immune-related disease. On the other hand, *IL7R* gene expression in blood is lower in older individuals, indicating that low *IL7R* gene expression might associate with reduced health. Interestingly, this contradictory result is supported by the observation that a higher *IL7R* gene expression level is associated with better prospective survival, both in the nonagenarians (Hazard ratio (HR) = 0.63, *P* = 0.037) and the middle-aged individuals (HR = 0.33, *P* = 1.9 × 10^–4^).

**Conclusions:**

Overall, we conclude that the IL7R network reflected by gene expression levels in blood may be involved in the rate of ageing and health status of elderly individuals.

**Electronic supplementary material:**

The online version of this article (doi:10.1186/s12979-015-0048-6) contains supplementary material, which is available to authorized users.

## Background

Ageing is the consequence of an accumulation of physiological changes over time, eventually increasing the mortality risk. Because ageing is the major risk factor for reduced wellbeing and for the most common human diseases of Western societies, treatment of elderly patients may be improved by understanding their biological age, the rate at which a person ages, and health status, rather than simply chronological age.

Many potential biomarkers of biological age have been suggested and are being tested for their association with chronological age, disease and prospective mortality [1]. In a search for new transcriptomic biomarkers of ageing, we found that the expression of 1853 genes in blood was associated with chronological age. Of these, 244 were associated with familial longevity, i.e. they were differentially expressed in middle-aged offspring of nonagenarian siblings as compared with controls from the Leiden Longevity Study (LLS). A low expression level of one of these 244 genes, the interleukin 7 (IL7) receptor (*IL7R*), associated with a better metabolic health profile [2]. Hence, *IL7R* gene expression in blood seems a good candidate biomarker for healthy ageing.

IL7R is important for the body’s innate and adaptive immune responses and plays a role in regulating development, differentiation and survival of T cells [3, 4]. IL7R is required for IL7 signalling, which in mice has been shown to be crucial for early T cell development, as well as for homeostasis of naïve and memory CD8+ T cells [5, 6]. Previously, we observed that the offspring of nonagenarian siblings avoid the usual age-related reduction of percentages and numbers of naïve T cells in the periphery [7]. Reduction of proinflammatory IL7 signalling may contribute to this better retention of naïve T cells and may thereby influence the biological age and health status of elderly individuals, given that possessing a fuller naive T cell repertoire would be expected to better protect against pathogens to which the individual has not been previously exposed.

To explore whether the expression profiles of other genes in the close vicinity of *IL7R* may exhibit even better ageing biomarker properties, we first identified the six interaction partners of *IL7R* using the STRING protein-protein-interaction database (http://string-db.org/). Next, gene expression levels of all seven genes in the *IL7R* gene network were measured in 87 nonagenarians who also had a nonagenarian sibling, 337 of their offspring, considered ‘healthy agers’, and 321 controls from the LLS. We tested whether the level of expression of the *IL7R* network genes associated with chronological age by comparing the nonagenarians with the middle-aged controls. Differential expression in this comparison may be explained by the age difference between these groups, early environmental factors or the longevity trait in these nonagenarians, which is not present in controls.

To further investigate whether the expression of the age-associated genes also associates with biological age and disease prevalence, we compared the offspring of the nonagenarians, representing individuals with a lower biological age, as marked by lower prevalence of age-related disease and beneficial metabolic profiles [8, 9], with similarly aged controls. Additionally, we explored whether the expression of the IL7R interaction partners is associated with common immune-related diseases in middle-aged individuals. Finally, we performed survival analysis in the nonagenarians and the combined group of middle-aged offspring and controls to determine the relationship between expression of these genes and mortality.

## Results

### IL7R network

**Fig. 1:**
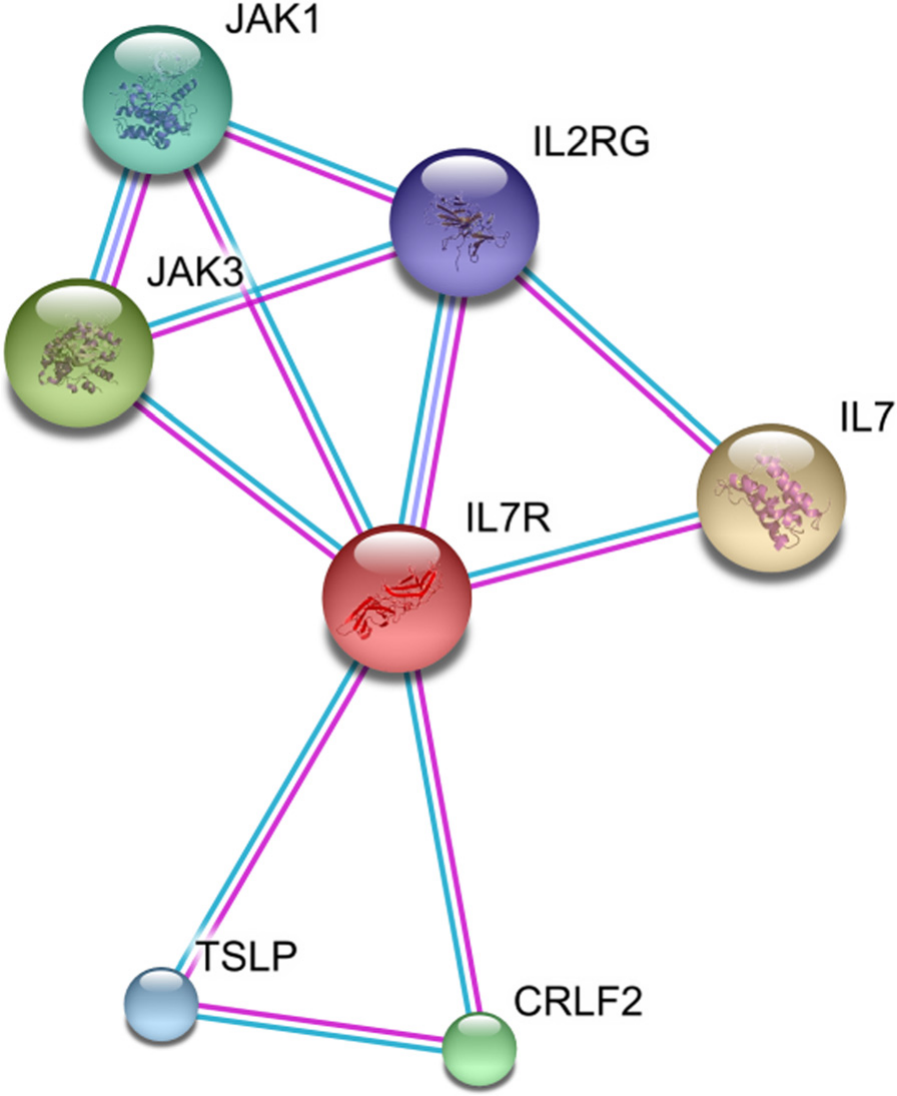
IL7R STRING network

To identify proteins interacting with IL7R, we searched for known and predicted protein-protein interactions using the STRING protein-protein-interaction database (http://string-db.org/) in May 2012. Interactions based on text mining were excluded, while predicted interactions based on experimental data with the highest confidence (score > 0.900) were taken into account. This approach resulted in an IL7R network that consists of IL7R and the following six interacting proteins: IL2RG, IL7, TSLP, CRLF2, JAK1 and JAK3 (Fig. 1).

### Gene expression in whole blood

We measured gene expression levels for each of the seven genes using RT-qPCR on whole blood samples from 87 nonagenarians, 337 of their middle-aged offspring and 321 middle-aged controls (Table 1). To test for associations between expression of genes in the IL7R network and chronological age, we compared the long-lived individuals with the middle-aged controls for differential expression of the total gene set of the IL7R network using a global test [10, 11], while adjusting for white blood cell counts. We observed that the expression level of the *IL7R* gene set as a whole was significantly different between the nonagenarians and younger controls (*P* = 4.6 × 10^−4^).

**Table 1:** Study characteristics

	Nonagenarians	Offspring	Controls
Number	87	337	321
Mean Age, years	94.3	61.3	61.2
Age Range, years	89.0–101.7	33.6–78.3	32.4–81.4
Women, N (%)	47 (54.0 %)	143 (42.4 %)	175 (54.5 %)
T2D, N (%)	NA	17 (5.0 %)	26 (8.1 %)
COPD, N (%)	NA	19 (5.6 %)	10 (3.1 %)
RA, N (%)	NA	3 (0.9 %)	2 (0.6 %)

*T2D* number of known patients with type 2 diabetes, *COPD* number of known patients with chronic obstructive pulmonary disease, *RA* number of known patients with rheumatoid arthritis, *NA* data not available

To investigate which genes were primarily responsible for the association of the IL7R network with age, we tested single gene expression levels using linear regression of the seven genes in the network (Table 2). After Bonferroni adjustment for multiple testing, three genes showed significant differential expression with at least a 5 % difference between nonagenarians and middle-aged controls, namely *IL7R*, *IL2RG* and *IL7*. We observed that expression of the IL7R complex/ligand genes *IL7R*, *IL2RG*, *IL7*, *TSLP* and *CRLF2* were all lower in nonagenarians, while expression of *JAK1* and *JAK3* was higher.

**Table 2:** Gene expression of nonagenarians compared to controls by linear regression analysis

Gene	Coef	FC	*P*	Bonferroni
***IL7R***	**−0.40**	**0.76**	**<10** ^**−6**^	**<0.001**
***IL2RG***	**−2.09**	**0.23**	**0.007**	**0.049**
***IL7***	**−0.81**	**0.57**	**<10** ^**−6**^	**<0.001**
*TSLP*	−0.01	0.99	0.013	0.091
*CRLF2*	−0.15	0.90	0.040	0.280
*JAK1*	0.21	1.16	0.046	0.322
*JAK3*	1.68	3.20	0.009	0.063

Genes significantly differentially expressed with at least 5 % are depicted in bold

*Coef* coefficient from linear regression model, *FC* fold change (above one indicates a higher expression in long-lived individuals), *P* raw *P*-value from the linear regression model, *Bonferroni P*-value after adjustment for multiple testing (N = 7) by the Bonferroni method

Because differential expression in these comparisons may be explained by the age difference between the two groups, cohort effects, or the longevity trait in these families, which is not present in controls, we investigated whether the differences in expression of the seven *IL7R* network genes was a characteristic of these long-lived families, exhibiting a lower biological age, and not just a marker for chronological age. Therefore, we compared the expression in the middle-aged offspring of nonagenarians to that in the similarly aged controls. Expression of the IL7R complex/ligands *IL7R*, *IL2RG*, *IL7*, *TSLP* and *JAK3* was found to be lower in offspring of nonagenarians as compared to controls. Of these, *IL7R* was significant after Bonferroni adjustment for multiple testing (Table 3), indicating its association with familial longevity and biological age. Similar results were obtained after adjustment for white blood cell counts (Additional file 1: Table S1). Expression of *JAK1* was higher in offspring as compared to controls. The direction of the differential gene expression was the same as for nonagenarians, except for *JAK3* for which the expression was lower in nonagenarians and higher in their offspring relative to controls.

**Table 3:** Gene expression of offspring from nonagenarians compared to controls by linear regression analysis

Gene	Coef	FC	*P*	Bonferroni
***IL7R***	**−0.18**	**0.89**	**0.001**	**0.006**
*IL2RG*	−0.72	0.61	0.563	1.000
*IL7*	−0.39	0.77	0.050	0.350
*TSLP*	−0.01	0.99	0.140	0.980
*CRLF2*	−0.04	0.97	0.742	1.000
*JAK1*	0.08	1.05	0.557	1.000
*JAK3*	−0.30	0.81	0.650	1.000

Genes significantly differentially expressed with at least 5 % are depicted in bold

*Coef* coefficient from linear regression model, *FC* fold change (above one indicates a higher expression in offspring from long-lived individuals), *P* raw *P*-value from the linear regression model, *Bonferroni P*-value after adjustment for multiple testing (*N* = 7) by the Bonferroni method

### Relation of *IL7R* gene expression in whole blood with membrane-bound IL7R protein in PBMCs

Since decreased *IL7R* gene expression associates with familial longevity already in middle-age, the question arises whether this reflects soluble or membrane-bound IL7R protein, also known as lymphocyte surface marker CD127. We were able to investigate membrane-bound CD127 levels in peripheral blood mononuclear cells (PBMCs) of 71 offspring of long-lived individuals and 73 controls. We observed no correlation between *IL7R* gene expression in whole blood and CD127 levels in PBMCs of the same individuals (Table 4, N_offspring_ = 53 and N_control_ = 53) and no difference in CD127 between the offspring and controls (mean level offspring = 38.1, mean level controls = 37.5, *P* = 0.91). Thus, rather than levels of membrane-bound CD127, it is more likely that the difference in gene expression levels between ‘healthy agers’ and controls reflect differences in levels of soluble CD127.

**Table 4:** Correlation of *IL7R *gene expression and CD127 expression in offspring from nonagenarians and controls

	Offspring (*n* = 53)		Controls (*n* = 53)	
Subset	Cor	*P*	Cor	*P*
PBMC	0.161	0.250	−0.171	0.221
Lymphocytes	0.169	0.226	−0.135	0.336
T cells	0.174	0.212	−0.159	0.256
Non-T cells	0.152	0.278	−0.130	0.353
CD4+ cells	0.169	0.227	−0.151	0.281
CD8+ cells	0.167	0.231	−0.135	0.336

*Cor* Pearson correlation, *P* raw *P*-value from Pearson correlation, *PBMC* peripheral blood mononuclear cells

### Association of *IL7R* gene expression with immune-related disease

Because IL7R and IL7 signalling have been implicated in the aetiology of immune-related disease [12–15], the observed differences in gene expression with biological age might also be associated with a different immune-related disease prevalence between the groups (Table 1). We investigated expression levels of *IL7R* relative to disease status for type 2 diabetes (T2D), chronic obstructive pulmonary disease (COPD) and rheumatoid arthritis (RA) in the offspring of long-lived individuals and controls. Table 5 shows that higher *IL7R* expression is associated with a higher prevalence of immune-related diseases. However, the difference in *IL7R* expression between offspring and controls remained after adjustment for prevalence of T2D, COPD and RA (Additional file 1: Table S2). Thus, the *IL7R* expression levels in blood associate with immune-related disease on the one hand and familial longevity on the other, independent of these immune-related diseases.

**Table 5:** Association of *IL7R *gene expression with immune-related diseases in middle-aged individuals

	T2D (N = 43)		COPD (N = 29)		RA (N = 5)		Sumscore (N = 70)	
Gene	Coef	*P*	Coef	*P*	Coef	*P*	Coef	*P*
*IL7R*	**0.22**	**0.029**	**0.35**	**9.9 × 10** ^**−5**^	−0.09	0.391	**0.21**	**0.001**

The numbers in the table represent the known numbers of patients, the total number of analysed individuals is 658. Significant associations are depicted in bold

*Sumscore* individuals with T2D, COPD, RA or a combination thereof, *Coef* coefficient from the linear regression model, *FC* fold change (above one indicates a positive association between gene expression and disease prevalence), *P* raw *P*-value from linear regression model

### Association of *IL7R *gene expression with mortality

**Fig. 2:**
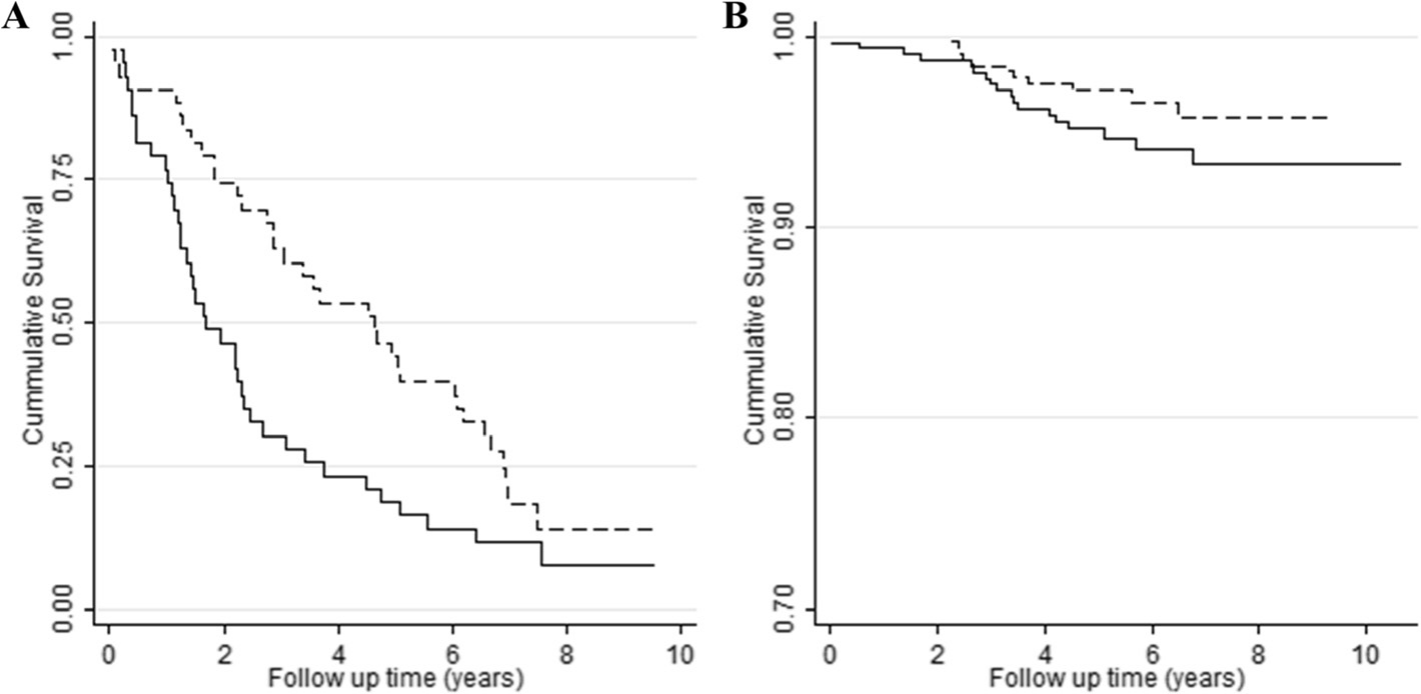
Kaplan-Meier curve for high (dotted line) and low (solid line) *IL7R* gene expression in 81 nonagenarians (**a**) and 619 middle-aged individuals (**b**)

Many markers indicating health status in middle age, such as blood pressure, also associate with mortality at higher ages, albeit not always in the expected direction [16]. We examined whether the *IL7R* gene expression level is associated with prospective mortality in the subset of LLS participants for which we have measured expression levels. We performed a survival analysis using a Cox proportional hazard model for low versus high gene expression levels in 81 nonagenarians (Fig. 2a) and the combined group of 619 of their middle-aged offspring and controls (Fig. 2b). Among the nonagenarians, as well as in middle age, high *IL7R* gene expression is associated with reduced mortality (Hazard ratio (HR) = 0.60, 95 % CI 0.40-0.92, *P* = 0.018; HR = 0.50, 95 % CI 0.30–0.82, *P* = 0.007). Thus, unexpectedly, higher *IL7R* gene expression levels in blood associates with better survival in both age groups.

## Discussion

In this paper we made two opposing observations. First, relative to controls, ‘healthy agers’, i.e. nonagenarian siblings and their offspring, were characterized by reduced *IL7R* expression in blood and increased *IL7R* expression in middle age is associated with higher prevalence of immune-related diseases. Second, since gene expression levels of *IL7R* decreases with chronological age, higher *IL7R* expression levels may indicate a more ‘youthful profile’ and thus better health, which was supported by the observation that higher *IL7R* gene expression levels, in both the nonagenarians and middle-aged individuals, associate with better prospective survival. The mechanism underlying these observations requires further investigation to determine whether regulation of *IL7R* gene expression has a causal role as determinant of the rate of ageing.

The notion that low *IL7R* expression levels are beneficial for reaching old age healthily corresponds with previous observations that autoimmune disease patients express increased levels of the IL receptor/ligand complex genes [12, 13, 15] and that antagonizing IL7 or IL7R may offer possible treatment [12, 14]. An increase in systemic inflammation has generally been reported with increasing age, so-called 'inflamm-ageing' [17]. Long-lived individuals, such as LLS nonagenarians, can be seen as ‘slow’ or ‘healthy agers’ who do not show the commonly observed age-related characteristics of 'immunosenescence' and display relatively low levels of proinflammatory markers [7]. Our results may suggest that nonagenarian members of long-lived families have more efficient IL7 signalling, since they seem to require less compensation because of their remaining naïve T cell population, resulting in greater reserve capacity to cope with infections in old age. On the other hand, very low IL7R signaling has been observed in severe combined immunodeficiency [18] and in HIV infection [19, 20]. Based on this, we conclude that, besides the troublesome effect of the absence of IL7 signalling, a somewhat lower baseline level of *IL7R* gene expression, and potentially IL7 signalling, may contribute to healthy ageing.

Despite these observations, the expression of the *IL7R* gene network was negatively correlated with age, suggesting that low expression levels correlate with decreased health, which is consistent with our observation that low *IL7R* gene expression associates with higher prospective mortality. Clearly, this finding is inconsistent with the observation that, in the same group of individuals, low *IL7R* gene expression level is associated with familial longevity and a lower prevalence of immune-related disease. A similar paradox has been found in PBMCs, where a lower frequency of naïve T cells (and a higher frequency of differentiated T cells) was associated with better survival in elderly individuals, while a higher frequency was found in offspring of nonagenarians compared to the controls, suggesting an association with lower biological age [7, 21]. This might be interpreted as follows; if the individual is able to counter the reduction of *IL7R* gene expression with age by keeping the level of differentiated T cells high as memory cells to control disease, this may be associated with healthy old age.

Contributing to the paradox, may be the fact that there are two forms of IL7R exerting different functions, membrane-bound and soluble IL7R. Membrane-bound IL7R, also known as lymphocyte surface marker CD127, may transduce IL7 signalling, while soluble IL7R may represent a negative compensatory mechanism regulating IL7 signalling [19, 22, 23]. Because we used the recommended Taqman assay for the measurement of *IL7R* gene expression that cannot distinguish between the *IL7R* splice forms, we are unable to interpret how IL7 signalling is affected by the gene expression changes. *IL7R* gene expression levels and CD127 did not correlate in the offspring or controls and CD127 did not show a difference between the two groups. The absence of correlation between *IL7R* gene expression levels and CD127 may be explained by the difference in cellular composition of whole blood and PBMCs, i.e. PBMCs do not contain basophils and eosinophils. However, a study by McKay and colleagues suggests that PBMCs are the main source for *IL7R* gene expression levels in whole blood [24]. In addition, adjustment for the different white blood cell counts results in similar associations in our whole blood gene expression analysis of *IL7R* (Additional file 1: Table S1), indicating that the white blood cell composition has limited effect on the expression of *IL7R* in our study. Although soluble IL7R has been shown to potentiate IL7 signalling [25], our results might suggest that the decrease in *IL7R* gene expression reflects mainly a decrease in the mRNA coding for soluble IL7R, resulting in more efficient IL7 signalling.

We showed that *IL7R* gene expression in blood is associated with immune-related disease. Previous meta-analysis and genome-wide association studies showed that genetic variation in the *IL7R* gene is associated with ulcerative colitis [26], multiple sclerosis [27–29], primary biliary cirrhosis [30] and type 1 diabetes [31]. These results may suggest that genetic variation in and the expression of the *IL7R* gene is involved in auto-immune and chronic inflammatory disease. Hence, optimal response of the immune system may contribute to human longevity.

Further evidence that IL7 signalling may contribute to biological ageing and longevity is that it is closely connected to mTOR signalling, a pathway known for its effects on lifespan in animal models, which has recently also been implicated in human ageing and longevity [32, 33]. Several studies in mice provided evidence for the connection between IL7 and mTOR signalling. IL7 induces phosphorylation of the mTOR complex 1 (mTORC1) downstream targets S6 and 4EBP1, an effect antagonized by the mTOR inhibitor rapamycin. The reverse may occur as well; rapamycin inhibits proliferation and induces apoptosis of pre-B acute lymphoblastic leukemia and these effects are abrogated by IL7 [34]. Functional studies have validated *IL7R* as a FoxO1 target gene [35]. Acute deletion of FoxO1 induced a rapid and profound downregulation of *IL7R* expression, which is associated with a significant reduction of *IL7R* mRNA [36]. In addition, cytokine stimulation (including IL7) induces FoxO1 phosphorylation and decreased transcription of target genes [36]. In B cells, it has been shown that the mTOR complex 2 (mTORC2) suppresses *IL7R* gene expression by regulating FoxO1 phosphorylation [37]. Taken together, decreased IL7 levels and decreased mTORC1 activity seem to go hand-in-hand, further implying that decreased IL7 levels are beneficial, since decreased mTORC1 increases lifespan. The same is true for decreased IL7R and increased mTORC2, the latter we have recently shown to be associated with human longevity [33]. Gene expression levels of several of the mTOR-related genes, including *RPTOR*, *FOXO1* and *MTOR*, are indeed positively and significantly correlated with *IL7R* expression in the LLS (data not shown). Considering the clear connections with IL7 signalling and similar findings on the level of gene expression variations, mTOR signalling might also be involved in “inflamm-ageing” as part of its lifespan-regulating effect.

In a previous studies of the LLS population, Dekker and colleagues found more apoptotic activity and less senescence in the cultured skin fibroblasts of the offspring as compared to controls [38]. In addition, flow cytometric analysis in HIV-infected individuals demonstrated that CD8+ cells expressing high levels of *IL7R* also expressed slightly higher levels of anti-apoptotic markers, whereas nearly all apoptotic cells had low levels of *IL7R* [39]. Our finding of decreased *IL7R* expression in members of long-lived families fits well with these previous results.

## Conclusions

An overall lower expression level in blood of genes belonging to the IL7R network was found to be associated with higher chronological age. Yet, low *IL7R* gene expression was significantly associated with familial longevity in middle-age, independent of white blood cell counts, while high *IL7R* gene expression was associated with an increased prevalence of T2D, COPD and RA. Intriguingly, nonetheless, higher *IL7R* gene expression associates with better prospective survival. The level of expression of the *IL7R* gene in blood is a very promising marker for healthy ageing in long - lived families, although further research is required to understand how *IL7R* gene regulation contributes to biological ageing.

## Methods

### Study population

#### Leiden Longevity Study

The individuals investigated in this study are participants of the LLS. The families participating in this study have at least two siblings with a minimum age for men of 89 years and for women of 91 years [40]. The offspring of these long-lived individuals were also included and, because they have an increased chance of becoming long-lived (30 % reduced standardized mortality rate) and a lower prevalence of age-related disease [9], we consider them ‘healthy agers’. In addition, the partners of the offspring, which are similarly aged and are subjected to the same environmental exposures, were included as population controls. Blood samples were taken from all the participants. The LLS was approved by the Medical Ethical Committee of the Leiden University Medical Center and all participants gave written informed consent.

### Sample collection and RNA preparation

For the current study, we selected 87 unrelated nonagenarians, 337 offspring and 321 partners belonging to 281 nuclear families (Table 1). These samples were randomly selected, but in such a way that age and gender were balanced between the groups and the age range was as large as possible. Only individuals without outlying cell counts (>3 SD below or above the standard error of the mean) were included. This subpopulation is representative for the whole LLS regarding disease prevalence and parameters involved in metabolic syndrome [9, 41]. From these non-fasted individuals, peripheral blood was harvested using PAXgeneTM tubes (Qiagen, Venlo, The Netherlands). The tubes were frozen and kept at −20 °C for ~3–5 years. After thawing at room temperature for at least 2 h, total RNA was extracted from the approximately 2.5 ml of peripheral blood in each tube following the manufacturer’s recommended protocol (PAXgene Blood RNA Kit Handbook, Qiagen, Venlo, The Netherlands). The quality and integrity of the total RNA was evaluated on the 2100 Bioanalyzer (Agilent Technologies, Amstelveen, The Netherlands) and the concentration was measured using a NanoDrop spectrophotometer (NanoDrop Technologies, Wilmington, DE, USA). Quality criteria included a 28S/18S ratio, as measured by the 2100 Bioanalyzer, of at least 1.2, and a total RNA yield of at least 3 μg.

### RT-qPCR

For all seven IL7R network genes the suggested Taqman® assay (Applied Biosystems, Bleiswijk, the Netherlands) was selected. Reverse transcription was performed with total RNA from blood of the 745 samples that passed QC using the First Strand cDNA Synthesis Kit, according to the manufacturer’s protocol (Roche Applied Science, Almere, the Netherlands). cDNA was amplified using the DNA Engine Tetrad® 2 Peltier Thermal Cycler (Bio-Rad, Veenendaal, The Netherlands). qPCR was then performed with the Taqman® method using the BiomarkTM 48.48 and 96.96 Dynamic Arrays (Fluidigm Amsterdam, The Netherlands). Relative gene expression of the BioMark™ Array data were calculated by using the 2^−ΔΔCt^ method, in which Ct indicates cycle threshold, the fractional cycle number where the fluorescent signal reaches the detection threshold [42]. *YKT6* was used as internal control and commercially available human total reference RNA (Clontech Laboratories, Mountain View, CA, USA) as reference RNA.

### White blood cell subtypes

In the whole blood samples of the participants the following white blood cell subtypes were counted using the automated Siemens ADVIA 1200 system (SMSD, Tarrytown, NY) in the Leiden Medical Diagnostics Center: leukocytes, thrombocytes, neutrophils, lymphocytes, monocytes, basophils and eosinophils.

### CD127 staining

Cryopreserved PBMCs were thawed and treated with human immunoglobulin (GAMUNEX; TalecrisBiotherapeuthics) and ethidium monoazide (EMA, Invitrogen) to block Fc receptors and stain dead cells followed by indirect staining for CD3 using an OKT3 supernatant and a Pacific Orange-conjugated anti-mouse IgG (Invitrogen). After blocking unbound secondary antibodies with mouse serum (Chemicon, Millipore), cells were surface-stained with CD4-Pacific Blue, CD127-Alexa Fluor 647 (BioLegend, San Diego, USA) and CD8-PerCP (BD Biosciences, Heidelberg, Germany). Cells were measured immediately using an LSR-II (BD).

For data analysis, EMA+ dead cells were excluded and lymphocytes were gated using an FSC vs. SSC dot plot based on their size and granularity. T cells and non-T cells were characterized as CD3+ and CD3− cells within the lymphocyte gate. In the CD3+ gate, CD4 and CD8 cells were characterized in a CD4 vs. CD8 dot plot as CD4 + CD8− and CD4−CD8+ cells, respectively. The mean fluorescence intensity (MFI) of Alexa-Fluor647 (CD127) was determined on total lymphocytes, CD3−, CD3+, CD4+ and CD8+ cells. To standardize for fluctuation of the instrument over the measurement period of a few weeks, MFI of each studied population was standardized against the MFI of an unstained control PBMC for each experimental day, by dividing the MFI of each population by that of the cells in the lymphocyte gate of the unstained control. Flow cytometry data analysis was performed using FlowJo software (Tristar, San Diego, USA).

### Statistical analysis

#### Geneset analysis of gene expression data

The Globaltest methodology was designed to determine whether the common expression pattern of genes within a pre-defined set is significantly related to clinical outcome [10, 11]. A generalized linear model is used to estimate a ‘Q-statistic’ for each gene set, which describes the correlation between gene expression profiles, X, and clinical outcomes, Y. The Q-statistic for a gene set is the average of the Q-statistics for each gene in the set. The Globaltest method was used to perform geneset analysis comparing two groups of individuals (either nonagenarians vs. controls or offspring vs. controls) including age (in offspring vs. controls only), gender and their interaction as covariates. The globaltest R package [43] has been used to perform analyses.

#### Single gene analysis of gene expression data

Differences in expression level between nonagenarians, their offspring and controls were assessed using linear regression. In these analyses, the expression level was the dependent variable and the two groups of individuals (either nonagenarians vs. controls or offspring vs. controls) were included in the model as a categorical variable together with age (in offspring vs. controls only), gender and their interaction as covariates. To take into account dependencies within sibships, robust standard errors were used, i.e. the variance was computed from the between family variation. The *P*-values were also based on these robust standard errors. Analyses were performed using the software package STATA/SE 11.0 (DPC Software, StataCorp 2009).

To further investigate the candidate genes, their expression level was associated with white blood cell counts. Subsequently, we adjusted both the comparison between nonagenarians and controls and between offspring and controls for each of these cell counts separately.

#### Association of gene expression with immune-related diseases

Information on medical history was requested from the participants’ general practitioners. Gene expression of *IL7R* and *IL7* was associated with prevalence of T2D, COPD and RA and the sum score of these diseases. The linear regression model with *IL7R*/*IL7* gene expression levels as outcome was adjusted for age, gender and their interaction. The sum score indicates the number of patients with T2D, COPD, RA or a combination thereof. Next, the comparison of *IL7R* and *IL7* gene expression between offspring and controls was adjusted for the prevalence of these diseases, using the linear regression model described above.

#### Prospective analyses

Prospective analyses of IL7R-related genes was performed with 81 nonagenarians and the combined group of 313 offspring and 306 controls. After a mean follow-up time of 7.40 (nonagenarians) and 6.15 years (offspring and controls), 82.7 % (N = 67, nonagenarians) and 6.5 % (N = 40, offspring and controls) of the individuals had died. Prospective analyses were performed using a left-truncated Cox proportional hazards model, to adjust for late entry into the dataset according to age. Age at inclusion, gender, their interaction (middle-age only), group (offspring or partner) and white blood cell counts were included as covariates. To take into account dependencies within sibships, robust standard errors were used, i.e. the variance was computed from the between family variation. The *P*-values were also based on these robust standard errors. Analyses were performed using the software package STATA/SE 11.0 (DPC Software, StataCorp 2009).

## Additional file

Additional file 1:
**Table S1.** Linear regression results of gene expression of offspring from long-lived individuals compared to controls, adjusted for white blood cell counts. **Table S2**. Linear regression results of *IL7R* gene expression of offspring from long-lived individuals compared to controls, adjusted for prevalence of immune-related diseases. (XLSX 12 kb)
